# Impact of childhood wheezing on lung function in adulthood: A meta-analysis

**DOI:** 10.1371/journal.pone.0192390

**Published:** 2018-02-02

**Authors:** Huan Ma, Yuanyuan Li, Lin Tang, Xin Peng, Lili Jiang, Jiao Wan, Fengtao Suo, Guangli Zhang, Zhengxiu Luo

**Affiliations:** 1 Chongqing Key Laboratory of Pediatrics, Chongqing, China; 2 Department of Respiratory Medicine, Children’s Hospital of Chongqing Medical University, Ministry of Education Key Laboratory of Child Development and Disorders, Chongqing, China; 3 China International Science and Technology Cooperation base of Child development and Critical Disorders, Chongqing, China; Universite de Bretagne Occidentale, FRANCE

## Abstract

**Background:**

A growing body of evidence shows that childhood wheezing may lead to recurrent or persistent symptoms in adulthood, such that persistent wheezing associated with lung function deficits often have their roots in the first few years of life.

**Objectives:**

We summarized information from several prospective cohort studies following children with or without wheezing into adulthood, to estimate the effect of childhood wheezing on adulthood lung function.

**Methods:**

Medical literatures were searched in the Medline, PubMed, ScienceDirect, Web of Science and Embase databases up to October 31, 2016. The adulthood lung function was selected as primary outcome, and chronic obstructive pulmonary disease (COPD) prevalence was selected as secondary outcome. The meta-analysis was performed with the Stata Version 14.0. A random-effects model was applied to estimate standardized mean difference (SMD) of lung function, and relative risk (RR) of COPD.

**Results:**

Six articles enrolling 1141 and 1005 children with and without wheezing, respectively. Meta-analysis showed that childhood wheezing decreased adulthood lung function as compared with no-wheezing subjects (SMD = -0.365, 95% confidence interval (CI): -0.569~-0.161, P = 0.000). Subgroup analyses indicated that childhood atopic wheezing reduced adulthood FEV1/FVC and FEV1%pred when compared with no-wheezing subjects. In addition, childhood atopic wheezing was significantly associated with COPD prevalence (RR = 5.307, 95% CI:1.033~27.271, P = 0.046).

**Conclusions:**

Our meta-analysis suggests that childhood wheezing may induce ongoing declined lung function that extends into adult life, as well as an increased risk of COPD prevalence when accompanied with atopy.

## Introduction

Wheezing is one of the commonest respiratory symptoms in children with a high incidence worldwide. It is manifested by the continuous whistling during breathing, resulting from narrowing or obstruction of the respiratory tract, the two most frequent causes of wheezing in children are clearly asthma and bronchiolitis, the less frequent causes include congenital anatomical abnormalities, aspiration of foreign bodies, and other pulmonary disorders[1]. Epidemiological survey data showed that about one-third of all children had at least one episode of wheezing before the age of three [2], and about half of all children wheezed at least once in the first six years of life [3]. The prevalence of childhood wheezing, especially childhood asthma, reflects a significantly increasing trend year by year [4,5]. Children with wheezing episode also presented with diminished airway function [6], which substantially impacted their quality of life and aggravated the financial burden of the family. In some low-income countries, childhood wheezing may be misdiagnosed, and may be the cause of significant morbidity and mortality [7]. Overall, childhood wheezing remains a widespread health concern worldwide. It has been long known that children with wheezing episodes are generally related to viral illness in the early life, and usually go on symptom remission with age; however, some cases would continue to have wheezing attacks and develop asthma [8]. A prospective study, that enrolled children with transient wheezing in early life and assessed pulmonary function at six years old, showed diminished airway function [3]. Another study traced wheezing children until they were 11 and 16 years old, and arrived at the same conclusion [9]. Moreover, there is evidence [10–12] that childhood wheezing may cause adulthood recurrent or persistent respiratory symptoms, such that persistent wheezing associated with lung function deficits commonly have their roots in the first few years of life. Furthermore, smoking is known to be a very important determinant for adult lung function and chronic obstructive pulmonary disease (COPD), but adult lung function decline could also be contributed by factors encountered early in life [13,14]. A previous review also demonstrated that developmental factors associated with multiple biologic mechanisms diminished airway function during the growing years, thus increasing the risk of COPD [15]. A cross-sectional study in Wellington aimed to investigate the association of various known risk factors for COPD, which suggested the strongest association between childhood asthma and COPD [16]. Based on the aforesaid, we pose two principal questions: First, did children with early childhood wheeze have diminished pulmonary function going into adulthood, and did infection-related and atopic wheezing (asthma) show the same effect on lung function? Second, is there any association between asthma and COPD prevalence in later life? To answer these questions, we collected information from several prospective studies [17–22] following children with or without wheezing into adulthood, selected the adulthood lung function as the primary outcome, and COPD prevalence as secondary outcome, and performed meta-analysis of the datasets. For our analysis, we defined wheezing children as those who had wheezing episode(s) during 0 (newborn) to 16 years of age.

## Methods

### Search strategy

Medical literatures were obtained by searching the following databases: PubMed, Medline, Science Direct, Web of Science and Embase up to 31st October, 2016. The search keywords were: (1) wheeze or wheezing; (2) children or childhood; (3) lung function or pulmonary function. Articles were acquired from PubMed using the following search terms: (wheeze [Title/Abstract] OR wheezing [Title/Abstract]) AND (children [Title/Abstract] OR childhood [Title/Abstract]) AND (lung function [Title/Abstract] OR pulmonary function [Title/Abstract]). Furthermore, additional references were identified from citations in the articles that were reviewed.

### Study selection criteria

The related literatures were evaluated by reviewing the titles and abstracts, and further assessed by reviewing the full texts. The studies selected for meta-analysis included those in which data collection began in the childhood, with long-term follow-up into adulthood, and with special focus on serial lung function data. We only included literatures meeting the following criteria: 1) study design: prospective studies must have followed children until 18 years old or above; 2) subjects: studies that included children as the majority of participants were included, for the purposes of the meta-analysis, and age was defined as ranging 0 to 16 years old; 3) wheezing: studies that selected children with wheezing as an observation group and children without respiratory symptoms as a control group; 4) outcome reporting: mean ($\overset{-}{X}$) and SD (95% confidence interval (CI) or SE) of adulthood lung function must be reported in the articles; 5) language: to be extracted, studies had to be in English.

Any literature was excluded if it belonged to: 1) review, case report, and meeting abstract; 2) repeat publication; 3) non-English article; 4) low quality literature.

### Data extraction and quality assessment

Two investigators (Yuanyuan Li, Lin Tang) independently extracted all data, and a third investigator (Xin Peng) settled the differences. The following information was extracted from each article: first author name, year of publication, country, sample size (those subjects that provided lung function data), age of subjects at baseline, duration of follow-up (years), wheeze patterns in each article, assessment method of wheeze, adulthood lung function index (if spirometry was performed in multiple periods, data of the final test was used for our analyses), and COPD definition. The Newcastle-Ottawa Scale (NOS) was used for the quality evaluation [23]. A article that met only one criteria in the scale could be awarded 1 point, a article meeting two criteria was awarded two points and so on. The total scores range from 0 to 9, scores of 0–5 and 6–9 were considered as low and high quality, respectively.

### Statistical analysis

The meta-analysis was conducted using the Stata Version 14.0. We used standardized mean difference (SMD) to estimate the pooled effect of childhood wheeze on adulthood lung function, and risk ratios (RR) to estimate the association between childhood atopic wheezing and COPD. Precision of the estimates was expressed as the 95% confidence interval (CI), and statistical significance was set at a P value < 0.05 for all analyses. The statistical heterogeneity was assessed using the Q and I^2^ statistics (significance was set at a P value <0.05 or I^2^>50%). A random-effects model was used when significant heterogeneity was observed; otherwise, a fixed effects model was utilized [24]. Furthermore, to explore the possible sources of heterogeneity in adult lung function, sensitive analysis and subgroup analysis were conducted. Publication bias was visually evaluated using funnel plots and statistically assessed using Egger’s and Begg’s tests and the ‘trim and fill’ method. A P value <0.05 was adopted as statistically significant.

## Results

### Literature selection

**Fig 1:**
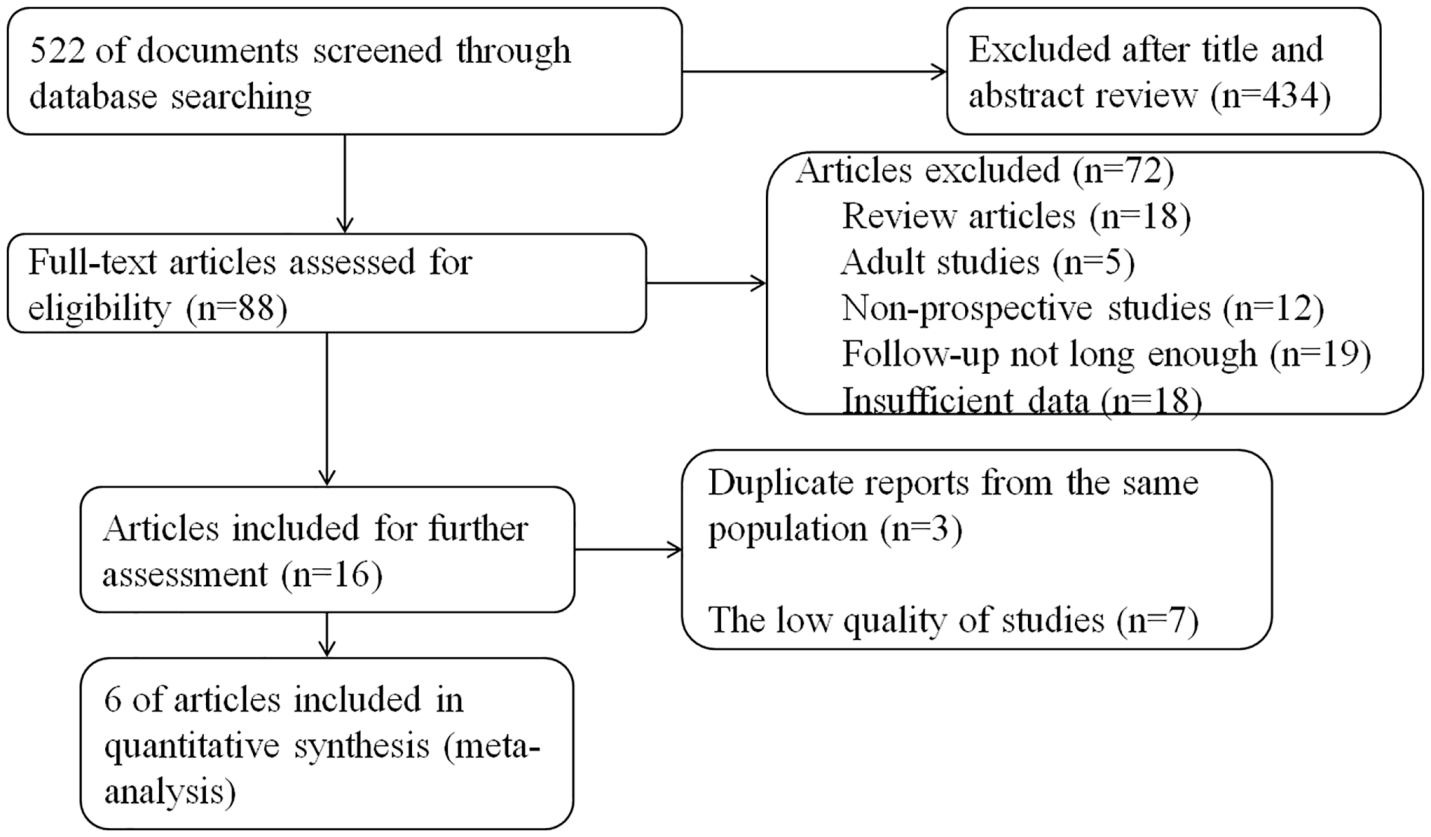
Flow chart of document screening and selection process.

We initially identified 522 references by electronic and manual search. After screening for the titles and abstracts, 434 records were excluded, and 88 full-text articles were selected. The reasons for further exclusion included: review articles (n = 18), adult studies (lacking children) (n = 5), non-prospective studies (n = 12), follow-up not long enough (n = 19), papers provided insufficient data (n = 18), duplicate reports from the same population (n = 3), and low quality literatures(n = 7). Six articles that fulfilled all the criteria were finally included (Fig 1).

### Study characteristics

The six included articles [17–22] were published from 2003 to 2016. They were published from Sweden and the USA (1 each), Australia and UK (2 each). Subjects were 1141 and 1005 children, with and without wheezing (control), respectively. The ages of these children ranged from 1 week to 15 years, and the duration of follow-up ranged from 18 to 50 years. Wheeze pattern in some articles differed from that in others, which defined from presence or absence of symptom, pathogenesis of wheeze, or age of wheeze onset. The wheeze was assessed in the beginning of the research, the methods were not quite consistent among these studies, including structured questionnaires or clinical assessment of physician. Wheeze assessment method was not provided in one article [22], which was acquired from previous studies with the same longitudinal cohort [25,26]. Adulthood lung function indices contained FEV1/FVC, FEV1%pred and Mean Change in FEV1 per year. The prevalence of COPD was provided in two articles[20,21], whereby COPD was defined according to the Global Initiative for Chronic Obstructive Lung Disease (GOLD) criteria: the diagnosis of COPD should be considered in any patient who has the following: symptoms of cough, sputum production, or dyspnoea, or history of exposure to risk factors for the disease; confirmed by spirometry: postbronchodilator FEV1/FVC less than 0.7[27,28]. The information of the included articles is summarized in Table 1.

**Table 1:** Information of enrolled prospective cohort studies.

Study (Pub year)	Country	Sample size	Age	Duration (years)	Wheeze pattern	Wheeze assessment	Pulmonary function
Sigurs N[17] (2010)	Sweden	138	< 1 yr	18	##(1) Transient, (2) Remission / intermittent, (3) Persistent / relapsing; (4) Late onset. Each pattern including two originally symptomatic groups 'asthma', 'recurrent wheeze'.	Asthma defined as ≥ 3 episodes of physician-verified wheeze, recurrent wheeze as 3 episodes of parent-reported wheeze.	FEV1/FVC (z-scores)
Stern DA[18] (2008)	USA	430	Infant	22	##(1) Inactive asthma, (2) chronic asthma. Two patterns including the originally symptomatic group 'asthma'.	Asthma defined as having ever had a physician diagnosis with active symptoms (attacks, episodes or wheeze).	FEV1/FVC (z-scores)
Horak E[19] (2003)	Australia	267	7 yr	35	#(1) Asthma, (2) severe asthma, (3) mild wheezy bronchitis, (4) wheezy bronchitis.	Asthma: children with wheezing not associated with respiratory tract infection, severe asthma: children with asthma plus barrel-chest deformity and/or FEV1/ FVC to <50%, mild wheezy bronchitis: children with <5 episodes of wheezing associated with bronchitis, wheezy bronchitis: children with <5 episodes of wheezing associated with bronchitis.	※FEV1/FVC, FEV1% pred
Tagiyeva N[20] (2016)*	UK	330	10–15 yr	50	#(1) Childhood asthma (CA), (2) WB/VAW (wheezy bronchitis/virus-associated wheeze).	After a pediatrician reviewed primary and secondary care medical records, and conducted a face-to-face clinical assessment.	※FEV1/FVC, FEV1% pred
Tai A[21] (2014)*	Australia	112	7 yr	43	##(1) Current asthma, including four originally symptomatic groups (WB-wheezy bronchitis, MWB-mild wheezybronchitis, asthma, SA-severe asthma).	WB- children with five or more episodes of wheezing associated with bronchitis, MWB-children with fewer than five episodes of wheezing associated with bronchitis, asthma-children with wheezing not associated with respiratory tract infection, SA-children with asthma combine with barrel-chest deformity and or FEV1/ FVC to 50% or less .	FEV1/FVC
Marossy AE[22] (2007)	UK	869	1 wk	45	#(1) Wheeze start at age 0 to 7, (2) wheeze start at age 8 to 16.	Structured questionnaires and examination by school medical officers.	Mean change in FEV1/yr

Notes:

* for COPD definition: according to the GOLD criteria: postbronchodilator FEV1/FVC<0.7;

^#^ for wheeze pattern definition: the wheeze pattern was classified in the beginning of the research and has been long in use till the end of follow-up;

^##^ for wheeze pattern definition: the wheeze pattern was reclassified at the end of follow-up, and included the originally symptomatic groups;

^※^ for lung function analyses: two lung function indices were provided in the study, we only chose FEV1/FVC to the lung function analyses, and added FEV1% pred to the subgroup analyses.

### Quality assessment

The scores of included articles ranged from six to eight (mean = 7, median = 7, SD = 0.894), which indicated the high quality of included studies and enhanced the reliability of the analysis (Table 2).

**Table 2:** Quality assessment of the included studies.

	Selection				Comparability		Outcome			Scores
Sigurs N [17]	*	*	*	×	*	*	*	*	*	8
Stern DA [18]	*	*	*	×	*	*	*	*	*	8
Horak E [19]	*	*	*	×	*	×	*	*	×	6
Tagiyeva N [20]	*	*	*	×	*	×	*	*	*	7
Tai A [21]	*	*	*	×	*	×	*	*	*	7
Marossy AE [22]	*	*	*	×	*	×	*	*	×	6

Notes: Studies that met the criteria were awarded 1 point for each asterisk; a missed criterion was marked as ×.

### Data analysis

#### Adulthood lung function

**Fig 2:**
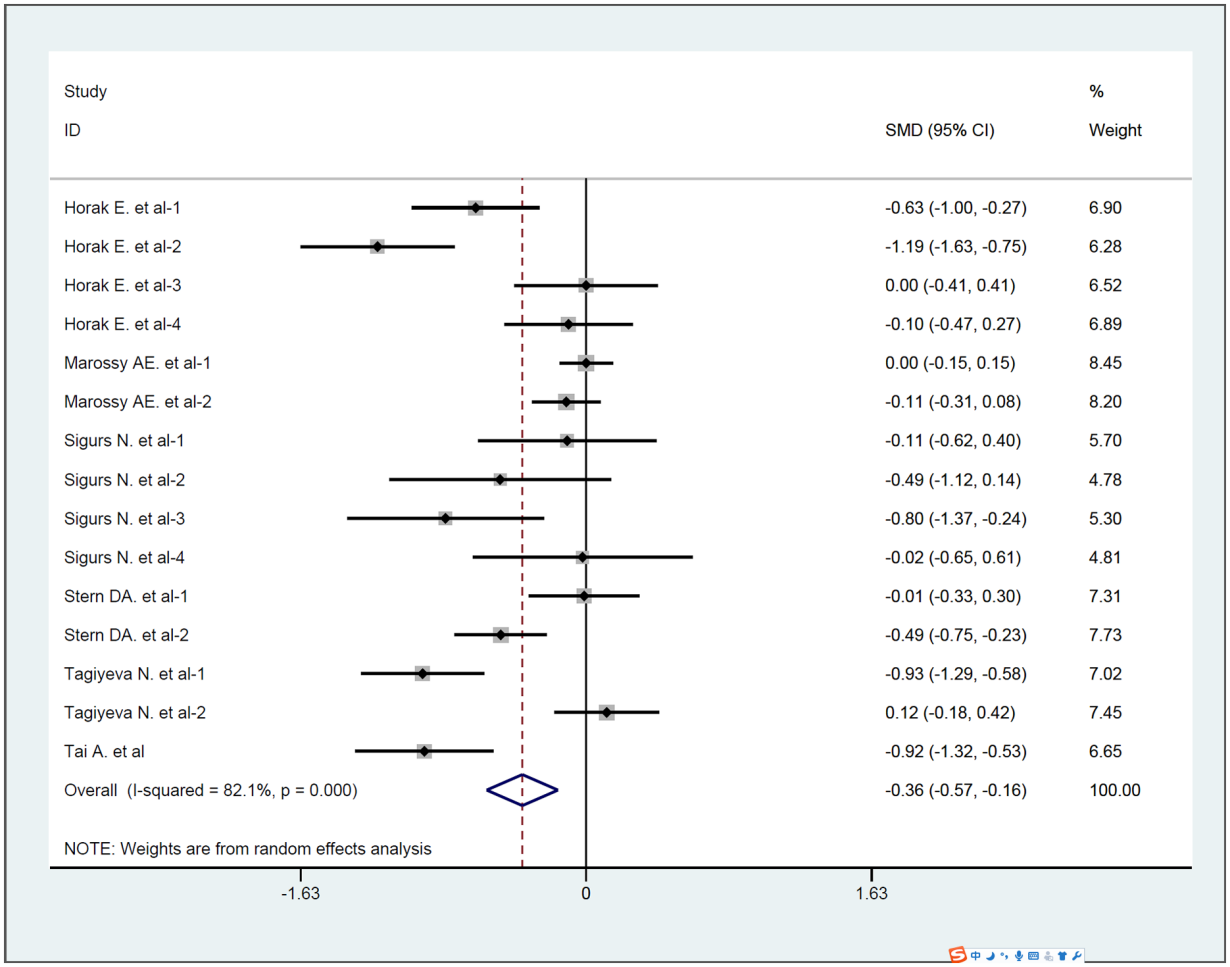
Pooled effect of childhood wheezing on adulthood lung function. Abbreviation: SMD = standardized mean difference, CI = confidence interval.

To estimate the effect of childhood wheezing on adulthood lung function, six [17–22] articles (15 prospective cohort studies) were pooled together in meta-analyses involving a total of 1141 subjects with wheezing and 1005 subjects without wheezing in childhood. Significant heterogeneity was detected among the included studies (chi-squared = 78.31, P = 0.000, I^2^ = 82.1%). Therefore, a random-effects model was used for the pooled analysis. The meta-analysis indicated that adulthood lung function was decreased in childhood wheezing group when compared with control group (SMD = -0.365, 95%CI: -0.569~-0.161, P = 0.000) (Fig 2).

### Sensitivity and subgroup analyses

**Fig 3:**
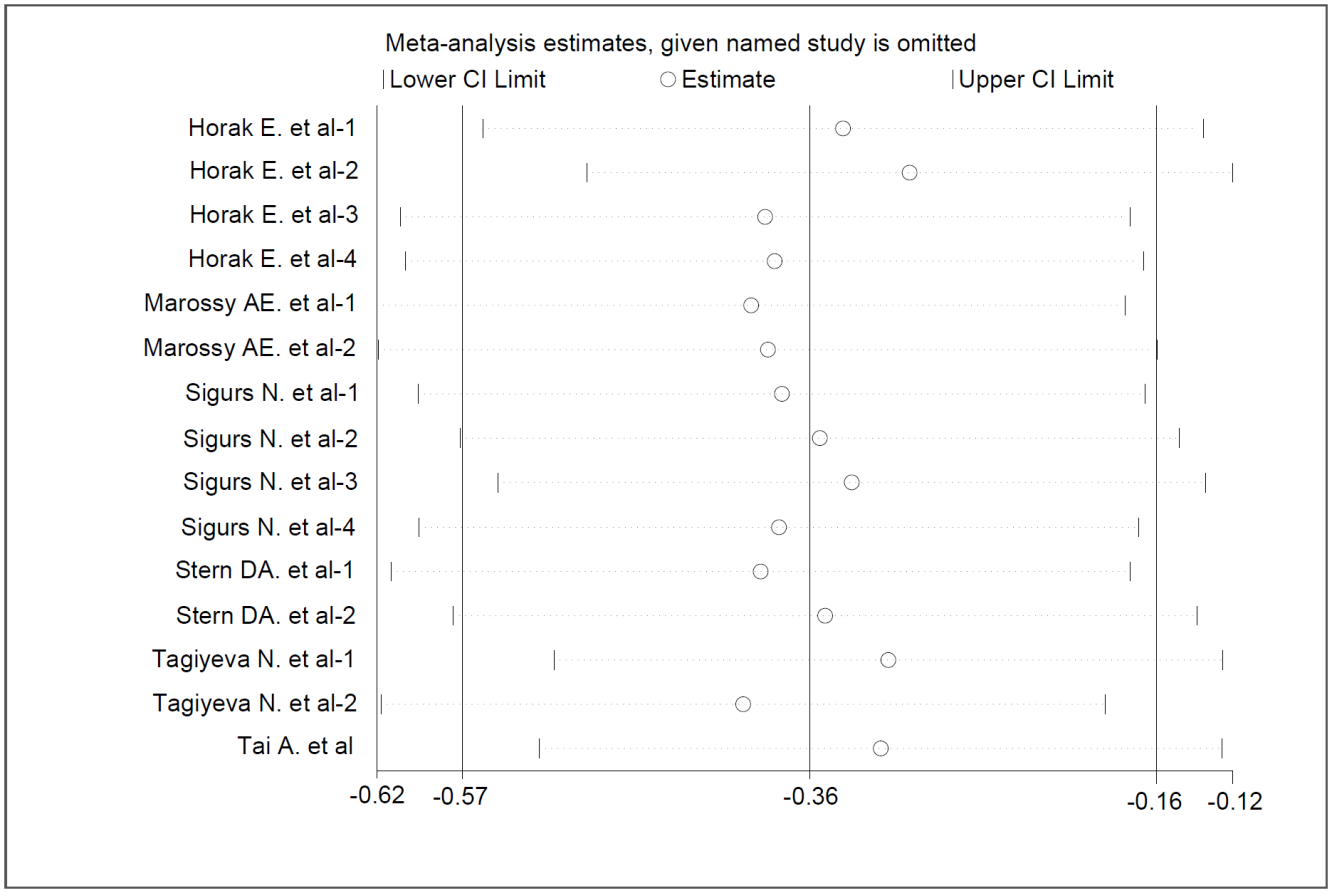
Sensitivity analysis of the included articles. Abbreviation: CI = confidence interval.

Sensitivity analysis showed that removal of each single study did not alter the overall results of pooled analyses (Fig 3). As shown in Table 1, the wheeze patterns of three articles [18–20] can be classified as atopic wheezing (asthma) and infection-related wheezing. Therefore, we carried out subgroup analyses to identify sources of heterogeneity and tried to minimize it, subgroups were stratified according to pathogenesis of wheeze and adulthood lung function indices (FEV1/FVC and FEV1%pred). We noted that FEV1/FVC (SMD = -0.635, 95% CI:-1.009~-0.260, P = 0.001) and FEV1%pred (SMD = -0.908, 95% CI:-1.128~-0.688, P = 0.000) were reduced in childhood atopic wheezing group when compared with control group. However, the FEV1/FVC (SMD = 0.024, 95%CI: -0.178~0.225, P = 0.817) and FEV1%pred (SMD = 0.050, 95% CI: -0.202~0.301, P = 0.697) were similar in childhood infection-related wheezing group and control group (Table 3).

**Table 3:** Subgroup analyses of two wheezing patterns (atopic and infection-related) and adulthood lung function.

Subgroups		N	Test for overall effect		Test for Heterogeneity		
			SMD (95%CI)	P	chi-squared	P	I^2^
Atopic wheezing	FEV1/FVC	3	-0.635(-1.009,-0.260)	0.001	24.40	0.000	83.6%
	FEV1%pred	2	-0.908(-1.128,-0.688)	0.000	1.53	0.465	0%
Infection related wheezing	FEV1/FVC	2	0.024(-0.178,0.225)	0.817	0.84	0.658	0%
	FEV1%pred	2	0.050(-0.202,0.301)	0.697	3.01	0.222	33.6%

Abbreviations and Notes: N, the number of articles; SMD, Standard Mean Difference; 95% CI, 95% confidence interval.

#### COPD prevalence

**Fig 4:**
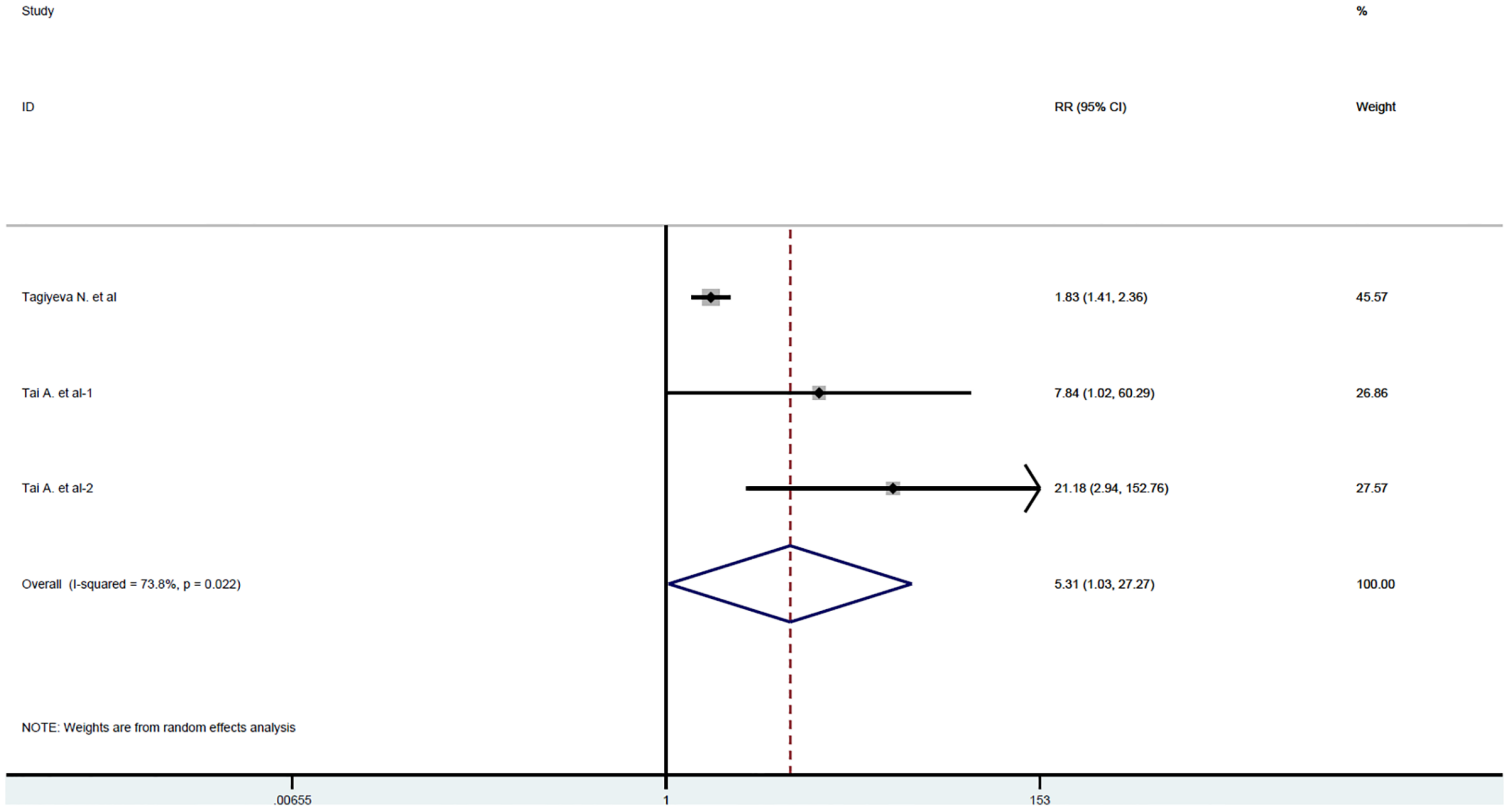
Pooled risk ratio for COPD with 95% confidence intervals of atopic wheezing children. Abbreviation: RR = risk ratios, CI = confidence interval.

Two articles [20,21] containing three studies provided COPD prevalence of childhood atopic wheezing and no wheezing subjects, age of those patients with COPD ranged from 50 to 65 years. Our meta-analysis indicated that childhood atopic wheezing was significantly associated with COPD prevalence (RR = 5.307, 95% CI:1.033~27.271, P = 0.046) with significant heterogeneity (chi-squared = 7.63, P = 0.022, I^2^ = 73.8%) (Fig 4).

### Publication bias

**Fig 5:**
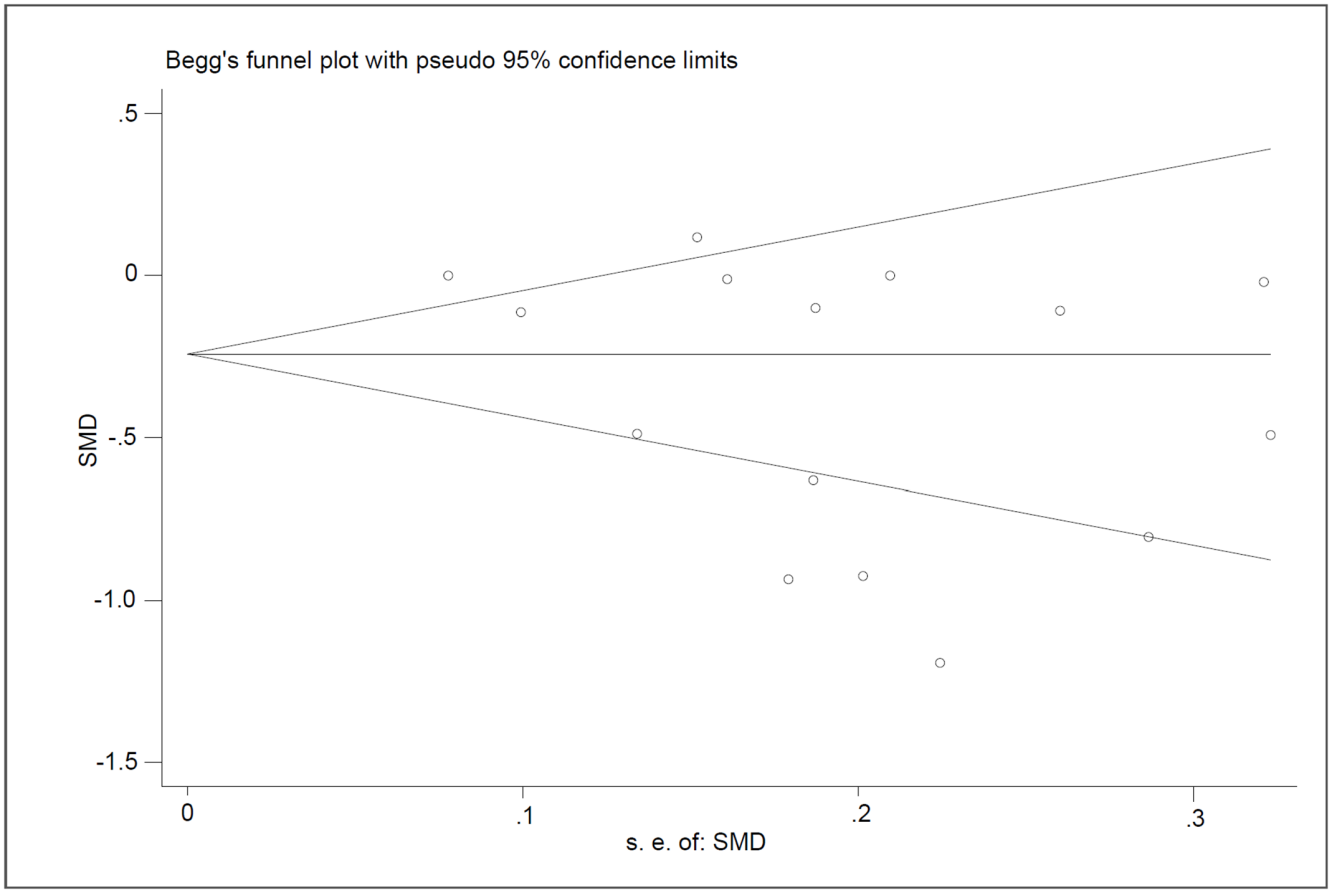
Funnel plots for assessing publication bias of the included studies. Abbreviation: SE = standard error, SMD = standardized mean difference.

There appeared to be funnel plot asymmetry for adulthood lung function (Fig 5), but the trim-and-fill method showed that no study needed to be statistically corrected for funnel plot asymmetry. The publication bias was not considered significant by using Begg’s (P = 0.113) and Egger’s (P = 0.063) tests.

## Discussion

Wheezing is one of the commonest respiratory symptoms in children with increasing prevalence in recent years. As mentioned, persistent wheezing with lung function deficits often have their roots in the first few years of life, and childhood atopic wheezing raises the risk of COPD in later life. The primary long-term outcome of this meta-analyses is adulthood lung function. 15 prospective cohort studies, which were analyzed, enrolled children with or without wheezing and followed them into various ages of adulthood. Our meta-analysis demonstrated that childhood wheezing decreased adulthood lung function, when compared with no-wheezing subjects. Two mechanisms of adulthood lower lung function were hypothesized, containing the initial damage sustained in early childhood and ongoing pathological progress of pulmonary parenchyma. Pulmonary disease in early life may interfere with lung and airway development [29]. The BAMSE cohort study [30] evaluated the lung function in adolescence based on different wheeze phenotypes, which found that the early transient wheeze group was associated with lower FEV1, FEV1/FVC, and FEF50 values compared with control group at 16 years of age. The same conclusion was indicated in the Environment and Childhood Asthma prospective birth cohort study [31]. These studies suggested that the initial damage sustained in early childhood is related to the consequent lung function decline in adolescence. There were conflicting outcomes of the early transient wheeze in adulthood. The Melbourne Atopy Cohort Study [32] reported that the early transient wheeze had a benign result with no sequelae for lung function impairment by age 18, which was in concordance with Tucson Children's Respiratory Study [18] and another prospective study in Sweden [17]. In addition, the persistent early wheezing was proven to be associated with lower lung function at both adolescence and adulthood in several studies [17,18,31,32]. Based on the overall consensus, we suggest that compared to the initial damage sustained in early childhood, the ongoing pathological progress of pulmonary parenchyma plays a larger role in lung function deficit into adult life.

Our subgroup analysis was based on the pathogenesis of wheezing (atopic or infection-related wheezing) and lung function index (FEV1/FVC, FEV1%pred). The results demonstrated that childhood atopic wheezing reduced adulthood FEV1/FVC and FEV1%pred when compared with no-wheezing subjects, while no significant differences were identified between the infection-related wheezing patients and controls. We presumed two possible explanations of this result. On the one hand, two published reviews suggested that early airway exposure of atopy was indicated as the most important risk factor to wheezing persistence [11,12]. The Childhood Asthma Management Program cohort[33] also reported that persistent atopic wheezing can result in abnormalities in FEV1/FVC decreasing and that these abnormalities increase in magnitude from ages 5 to 18 years. So we presume that this result is due to the relatively favourable prognosis of childhood infection-related wheezing, when compared with atopic wheezing [34]; on the other hand, the subgroup analysis only contained two articles to evaluate the effect of childhood infection-related wheezing on adulthood lung function, so the limited statistical power might be another possibility. Thus, there is insufficient evidence to prove that childhood infection-related wheezing is associated with reduced lung function in adulthood, and that further research is needed in this area.

Lastly, the BAMSE cohort [30] indicated that childhood asthma is associated with an overall increase in airway resistance. Several cross-sectional studies and reviews [35–40] demonstrated that childhood atopic wheezing emerged with a positive association for COPD. Thus, we further evaluated the effect of the childhood atopic wheezing on COPD prevalence based on three prospective studies[20,21]. Our meta-analysis found that childhood atopic wheezing increased the risk of COPD prevalence. Airway remodelling, as the consequence of ongoing pathological progress of pulmonary parenchyma, which is characterized by subepithelial fibrosis, reticular basement membrane (RBM) thickening, increased angiogenesis and airway smooth muscle (ASM) hypertrophy, is considered as the main reason for the lower adulthood lung function and high risk of COPD in atopic wheezing children [41]. Evidence has shown that incipient airway remodelling can be observed in preschool children with wheezing [42]. All of the pathologic changes of tracheobronchial tree, as previously described, are caused by different immunological or cellular mechanisms. Transforming growth factor and epidermal growth factor play critical roles in increasing fibroblasts, eventually leading to subepithelial fibrosis [43,44]. Moreover, extracellular matrix overproduction also contribute to fibrosis [45]. Keglowich et al [45] demonstrated that the thickness of the RBM in adult asthmatics increased up to 5 times as compared to healthy, and this phenomenon was also found in asthmatic children [46,47]. The deposition of collagen I and III, tenascin, and fibronectin, considered to be produced by activated myofibroblasts, contribute to RBM thickening [43]. In addition, several angiogenic factors secreted by ASM cells, such as vascular endothelial growth factors, participate in the increased vascular permeability and angiogenesis of submucosa, leading to airway edema and smooth muscle proliferation, and eventually resulting in ASM hypertrophy [45, 48]. Indeed, airway inflammatory cells (mainly eosinophils and mast cells) are shown to secrete angiogenic factors as well, which resulted in angiogenesis, linked to airway bronchial hyperresponsiveness (AHR) [49,50]. Other lines of evidence also showed that AHR may increase compressive mechanical stress and activate airway repair, and may have stronger effects on airway structure during early childhood, thus contributing to remodeling mechanisms, independent of airway inflammation [15]. Therefore, both the airway remodeling and AHR in atopic wheezing children are definitive reasons of the airflow obstruction, leading to lung function decline and the high risk of COPD, as observed. In addition, specific toxic proteins of the respiratory tract, such as eosinophil cationic protein, which are released by eosinophilic granulocyte, cause airway mucosa damage, and constitute another important determinant for the development of COPD. It is also possible that additional factors, such as smoking, coinheritance of genes or low socioeconomic status, partially confound the association between childhood atopic wheezing and COPD. To sum up, childhood atopic wheezing is associated with early airway dysfunction (combining airway inflammation and airway remodelling) due to diverse physiopathologic mechanisms, the resultant effect is diminished lung function in adult life and an increased risk factor of COPD.

### Strengths and limitations

To the best of our knowledge, this is the first meta-analysis to estimate the effects of childhood wheezing on adulthood lung function. Our meta-analysis was rigorously performed and reported according to the PRISMA checklist. A highly sensitive and comprehensive search of the literature was used in order to identify as many relevant studies as possible and to reduce potential publication bias. We have exclusively collected the studies of prospective design and of high quality. It demonstrated that the childhood wheezing accompany with atopy, as manifested by early airway dysfunction, is associated with progressive lung function decline into adult life and an increased risk of COPD. Nonetheless, our analysis has several unavoidable limitations. The first one is the small number of included articles and the high heterogeneity of lung function, despite our best efforts to retrieve all related data. This is because most literatures failed to meet our stringent inclusion criteria. The high heterogeneity may result from the different degrees of severity of wheezing and huge range of age (from 1 week to 15 years). Unfortunately, the kind of details needed for stratifying the data into further subgroups were not provided in the original studies. Additionally, subjects with COPD in our collected publications were relatively few, also relatively young, ranging from 50 to 65 years old, whereas COPD often develops later in life, and thus, we may have missed potential would-be COPD patients. Clearly, further research, comprising of larger cohort size, more detailed age-related data, and inclusion of older people in longitudinal studies relating childhood wheezing with COPD, need to be undertaken.

## Conclusion

In general, this meta-analysis suggests that childhood wheezing may induce ongoing declined lung function that extends into adult life, as well as an increased risk of COPD prevalence when accompanied with atopy.

## Supporting information

S1 Table Available data set. (XLSX)

S1 File PRISMA checklist. (DOC)
